# Association between maternal hemoglobin concentration levels and preterm birth clinical subtypes: A retrospective, observational, multicenter study

**DOI:** 10.1371/journal.pone.0348071

**Published:** 2026-05-13

**Authors:** Peiran Chen, Yi Mu, Yanxia Xie, Yanping Wang, Zheng Liu, Mingrong Li, Juan Liang, Jun Zhu

**Affiliations:** 1 National Office for Maternal and Child Health Surveillance of China, West China Second University Hospital, Sichuan University, Chengdu, Sichuan, China; 2 Department of Obstetrics, West China Second University Hospital, Sichuan University, Chengdu, Sichuan, China; 3 Key Laboratory of Birth Defects and Related Diseases of Women and Children (Sichuan University), Ministry of Education, Chengdu, China; Xiangya Hospital Central South University, CHINA

## Abstract

**Background:**

As a routine component of antenatal care, hemoglobin (Hb) testing can detect underlying abnormalities in pregnant women, thereby guiding clinical interventions. However, Hb concentration levels and trajectories during pregnancy and the association with preterm birth remain contentious owing to the unclear mechanisms involved. We aimed to evaluate the association between Hb concentration levels and preterm birth clinical subtypes in each trimester, and to assess the association between Hb trajectories and preterm birth clinical subtypes in pregnancy.

**Methods:**

This is a secondary analysis of a previously conducted cohort which is a retrospective, observational, multicenter study. The study included women with singleton live births between 28 and 45 gestational weeks from 2012 to 2021 who had undergone at least one Hb test before 37 weeks. Poisson regression with a robust variance estimator was used to estimate the association between preterm birth and Hb concentration levels measured at a given time. We included women from the overall sample who initiated antenatal care before 14 weeks and who had at least one Hb test in each trimester for analysis of their Hb trajectory. Multivariable logistic regression was used to estimate the association between preterm birth and Hb trajectories. Outcome measurements included preterm birth and clinical subtypes.

**Results:**

We included 72,139 women in this analysis, the preterm rate, spontaneous preterm rate and iatrogenic preterm rate were 6.73%, 4.12% and 2.50% respectively. In relation to an Hb cutoff value of 110 g/L in the third trimester, a “L” shape of curve was observed in the association between spontaneous preterm and Hb concentration, and a “U” shape of curve was observed in the association between preterm, iatrogenic preterm and Hb concentration. 17,101 women were enrolled in the trajectory analysis. A constant high Hb concentration trajectory was associated with an increased risk of preterm and iatrogenic preterm (odds ratio with its 95% Confidence Interval, OR[95%CI] were 1.22[1.05,1.42] and 1.35[1.03,1.78] respectively), and a constant low Hb concentration trajectory was associated with an increased risk of spontaneous preterm (OR[95%CI], 1.30[1.03,1.64]).

**Conclusion:**

The association between preterm birth and both low and high Hb levels should not be ignored. Women with anemia in late pregnancy should be aware of iatrogenic and spontaneous preterm birth, and women with high Hb concentrations should be aware of iatrogenic preterm birth. Constantly high Hb level may warrant an alert for iatrogenic preterm birth, and constantly low Hb level may warrant an alert for spontaneous preterm birth.

## Introduction

The hemoglobin (Hb) test forms part of routine antenatal care and is performed several times during pregnancy, the abnormal Hb concentration level may indicate adverse outcomes of pregnancy [1]. The low Hb concentration level such as anemia (<110 g/L) is a common maternal complication during pregnancy, with moderate and severe anemia defined as Hb levels <99 g/L and <70 g/L, respectively [2]. It has been reported that moderate or severe anemia is associated with an increased risk of maternal shock, intensive care unit (ICU) admission, maternal death, fetal growth restriction, and stillbirth [3]. Consequently, the adverse effects of a low Hb concentration level seems to have drawn more attention than those of a high Hb concentration, and few studies have investigated the association between high Hb concentration levels and adverse perinatal outcomes. In fact, regarding preterm birth, while there are conflicting conclusions, some studies have reported that both low and high Hb levels or increased Hb levels in late pregnancy are associated with an increased risk of preterm birth [4–8]. Preterm birth can be categorized into specific clinical subtypes, such as spontaneous and iatrogenic preterm birth, based on different obstetric precursors [9]. Iatrogenic preterm births included those who underwent labor induction or caesarean delivery for maternal or fetal indications [9]. Maternal indications included complications such as hypertensive disorders, diabetes, placental abruption, obstetric hemorrhage, abnormal amniotic fluid, acute appendicitis, cardiovascular disease, liver disease, and intrahepatic cholestasis during pregnancy. Fetal indications included fetal distress in the uterus and fetal growth restriction [10]. Despite different potential mechanisms, few studies have investigated the association between Hb concentration and the risk of specific clinical subtypes of preterm birth [7].

Hb concentrations are known to fluctuate throughout pregnancy. Normal physiological hemodilution during early pregnancy may lead to a decrease in Hb concentration from the first trimester. However, whether the change trace of Hb in all trimesters follows a “U” shape or an “L” shape remains contentious [7,11–14]. With conflicting conclusions concerning the association between anemia and preterm birth at a point in time measure of Hb concentration in most studies, the association between anemia and preterm birth has remained unclear. Therefore, assessing the longitudinal trajectory of Hb concentration during pregnancy and its association with preterm birth may help clarify this relationship [5,7].

We aimed to determine the association between Hb concentration and preterm birth and its clinical subtypes in separate trimesters, and to analyze the trajectory of Hb concentration and its association with preterm birth. Longitudinal Hb values were obtained from a cohort of pregnant women who received antenatal care before 14 weeks of gestation and who had routinely received antenatal care in each trimester.

## Methods

### Study design and participants

This is a secondary analysis of a study aimed at providing a data foundation for research related to birth defects. Data were collected from a multicenter cohort, this cohort includes ten hospitals located in eastern (4 hospitals), central (5 hospitals), and western (1 hospital) regions of China. This cohort retrospectively collected data on pre-pregnancy, prenatal and postnatal laboratory tests, socio-demographic characteristics and pregnancy outcomes of hospitalized delivery pregnant women. This study retrospectively collected demographic characteristics of women from electronic health records; laboratory examination data in relation to routine antenatal care, such as Hb concentration tests (in g/L); the laboratory information system; and perinatal records from the health information system during pregnancy and delivery. Information from each system was connected based on the maternal identity number. Data were obtained on 15^th^ July 2022 for research analysis, and the data were de-identified prior to analysis.

**Fig 1:**
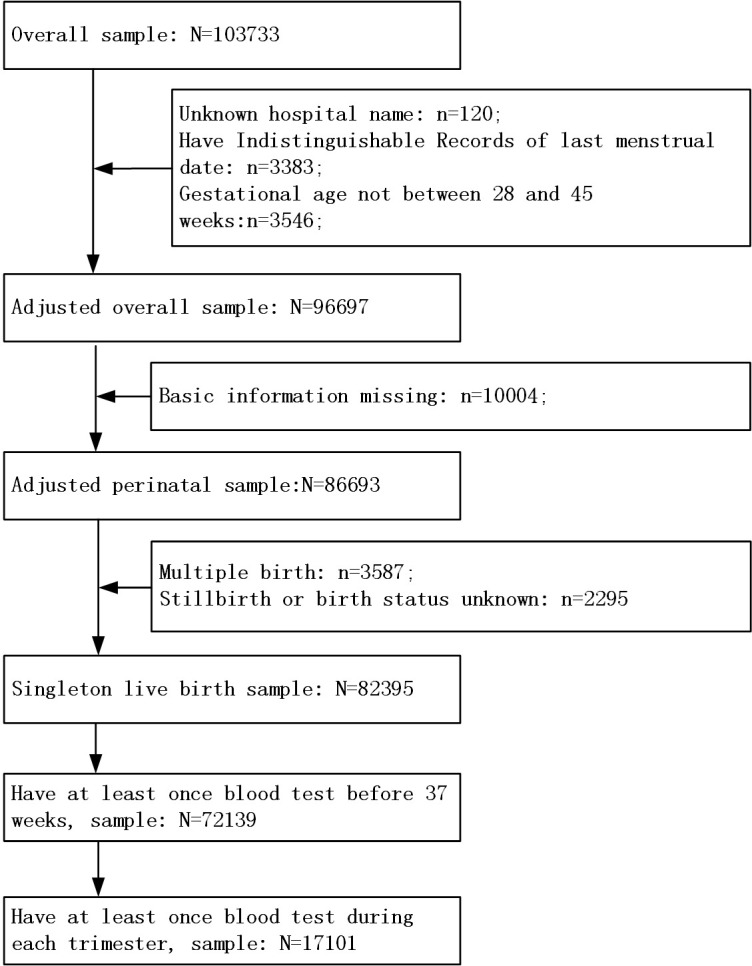
Flow chart of participants in the study.

We restricted the analysis to determining the association between maternal Hb concentration levels and preterm birth in women who delivered singleton live births between 28 and 45 weeks of gestation and had only one pregnancy record between 2012 and 2021. In China, the definition of a perinatal infant includes those with a gestational age of 28 weeks or more or a birth weight of over 1000 grams. Therefore, in this study, the gestational age of the neonates was defined as 28 weeks or more. We excluded women who did not have Hb test results before 37 weeks of gestational age. In addition, we restricted the study population to women who initiated antenatal care before 14 weeks and who had undergone at least one Hb test in each trimester to analyze their Hb trajectories. Details of the inclusion and exclusion criteria are presented in a flowchart in **Fig 1**.

### Outcomes

Preterm birth was defined as a single live born infant born at gestational age >=28 weeks and <37 weeks. The gestational age was determined using ultrasound dating. Preterm births were categorized as either spontaneous or iatrogenic based on their ICD-10 code (“O60.1” for spontaneous preterm and “O60.3” for iatrogenic preterm). Spontaneous preterm births included those with spontaneous contraction onset or rupture of membranes before 37 weeks of gestation.

### Covariates

This study was adjusted for the following covariates related to preterm birth: (i) demographic factors such as maternal nationality, age, work, educational level, parity, and previous medical history; (ii) reproductive history of preterm birth and scarred uterus; and (iii) medical factors for the present pregnancy such as fetal sex, use of assisted reproduction technology, and cervical incompetence.

### Statistical analysis

Descriptive analyses were used to summarize maternal demographic characteristics, previous medical and reproductive history, and characteristics of the present pregnancy, using frequencies and percentages for categorical data and medians (interquartile range [IQR]) for continuous variables.

We adopted restricted cubic splines to fit the nonlinear association between Hb concentration and preterm birth. We set five knots (5th, 27.5th, 50th, 72.5th, and 95th percentiles) for the models. To estimate the risk of preterm birth using separate Hb concentration values, we used 110 g/L as a reference in a Poisson regression model with a robust variance estimator, considering the cluster effect of hospitals and pregnant women [6,15].

We further analyzed the trajectory of Hb concentrations among women who had at least one Hb test result in each trimester. Mean Hb concentration values of each enrolled woman by trimester were fitted using a group-based trajectory model. We only had three time points; therefore, it was difficult to set the trajectory shape of each group as a cubic order. We started with all groups set to have a linear order and then compared changes in Bayesian information criteria (BIC), and the significance of parameters as their orders were made more complex in a quadratic order. The models were fitted with a normal distribution of Hb concentrations ranging from two to five trajectories. The most optimal pattern in the trajectories was selected using the following criteria: (i) the lower absolute value of BIC of the model; (ii) the average posterior probability of each group was ≥ 0.7; and (iii) the sample size of each group was > 5% of the total participants. [16] Details of the optimal trajectory pattern selection are listed in **Supplementary Table 3 in** S1 File. Multivariable logistic regression models adjusted for covariates were used to compare the association between different Hb concentration trajectory groups and preterm birth. Multivariate logistic regression models adjusted for covariates were used to compare the association between different Hb concentration trajectory groups and clinical subtypes of preterm birth. Sample size of each Hb concentration trajectory group in the overall analysis and different subgroup analysis was showed in **Supplementary Table 6** in S1 File.

Maternal nationality, work, and education are unstructured data, making it difficult to extract them using software. Moreover, some hospitals did not strictly collect the above information when admitting pregnant women and mothers, resulting in significant proportions of missing data. To verify the impact of the missing data on the analysis results, multiple imputation was used for missing data as the sensitivity analysis. Details of unknown data and sensitivity analysis were shown in **Supplementary Table 1**, **Supplementary Figure 1** and **Supplementary Table 4** in S1 File.

All statistical analyses were conducted using two-tailed tests with a significance level of *P* < 0.05. All analyses were performed using SAS (version 9.4; SAS Institute Inc., Cary, NC, USA), Stata version 15.1 (Stata Corp., TX, USA) software, and R version 4.3.0 (R Foundation for Statistical Computing, http://www.r-project.org) software.

### Ethics approval and consent to participate

This study was approved by the ethics committee of the West China Second University Hospital (protocol ID,2019088) in accordance with the Declaration of Helsinki. All participants provided written informed consent.

## Results

In total, 72,139 women were included in this analysis (**Fig 1**). Of the enrolled women, 67,286 women delivered term births, and 4,853 (6.73%) women delivered preterm births, including 2,969 (4.12%) spontaneous preterm and 1,803 (2.50%) iatrogenic preterm births. Compared with term birth women, preterm birth women had a higher proportion of advanced maternal age (24.46% vs. 16.45%), multipara (49.45% vs. 37.56%), preterm history (4.47% vs. 0.99%) and a scarred uterus (28.91% vs. 17.38%), respectively (**Table 1**).

**Table 1:** Baseline character for all enrolled pregnant women. (N = 72139).

Character	Term birth	Preterm birth	Subtype of preterm birth*	
			Spontaneous preterm	Iatrogenic preterm
	N = 67286	N = 4853	N = 2969	N = 1807
**Maternal age**				
<35 yrs	56200(83.52%)	3659(75.40%)	2299(77.43%)	1301(72.16%)
≥35 yrs	11070(16.45%)	1187(24.46%)	669(22.53%)	498(27.62%)
Unknown	16(0.02%)	7(0.14%)	1(0.03%)	4(0.22%)
**Parity**				
Primipara	41769(62.08%)	2413(49.72%)	1594(53.69%)	795(44.09%)
Multipara	25274(37.56%)	2400(49.45%)	1351(45.50%)	999(55.41%)
Unknown	243(0.36%)	40(0.82%)	24(0.81%)	9(0.50%)
**Medical history**				
None	56946(84.63%)	4036(83.17%)	2499(84.17%)	1475(81.81%)
At least one	10338(15.36%)	815(16.79%)	470(15.83%)	328(18.19%)
Unknown	2(0.00%)	2(0.04%)	–	–
**Preterm history**				
None	65820(97.82%)	4560(93.96%)	2794(94.11%)	1694(93.95%)
Yes	668(0.99%)	217(4.47%)	134(4.51%)	80(4.44%)
Unknown	798(1.19%)	76(1.57%)	41(1.38%)	29(1.61%)
**Scarred uterus**				
No	55577(82.60%)	3446(71.01%)	2305(77.64%)	1087(60.29%)
Yes	11691(17.38%)	1403(28.91%)	664(22.36%)	716(39.71%)
Unknown	18(0.03%)	4(0.08%)	–	–
**Fetal gender**				
Boy	34602(51.43%)	2726(56.17%)	1707(57.49%)	976(54.13%)
Girl	32320(48.03%)	1998(41.17%)	1193(40.18%)	776(43.04%)
Unknown	364(0.54%)	129(2.66%)	69(2.32%)	51(2.83%)
**Assisted reproduction**				
No	64624(96.04%)	4601(94.81%)	2815(94.81%)	1711(94.90%)
Yes	2644(3.93%)	248(5.11%)	154(5.19%)	92(5.10%)
Unknown	18(0.03%)	4(0.08%)	–	–
**Cervical incompetence**				
No	67018(99.60%)	4754(97.96%)	2907(97.91%)	1775(98.45%)
Yes	250(0.37%)	95(1.96%)	62(2.09%)	28(1.55%)
Unknown	18(0.03%)	4(0.08%)	–	–
**Pre-eclampsia**				
No	65803(97.80%)	3968(81.76%)	2804(94.44%)	1091(60.38%)
Yes	1465(2.18%)	881(18.15%)	165(5.56%)	716(39.62%)
Unknown	18(0.03%)	4(0.08%)	–	–
**Gestational hypertension**				
No	65370(97.15%)	4689(96.62%)	2881(97.04%)	1735(96.02%)
Yes	1898(2.82%)	160(3.30%)	88(2.96%)	72(3.98%)
Unknown	18(0.03%)	4(0.08%)	–	–
**Gestational diabetes**				
No	52126(77.47%)	3619(74.57%)	2178(73.36%)	1369(75.76%)
Yes	15142(22.50%)	1230(25.35%)	791(26.64%)	438(24.24%)
Unknown	18(0.03%)	4(0.08%)	–	–
**Hemoglobin concentration (g/L)**				
Trimester 1 Median [Q1, Q3]	127.0[121.0,133.0]	128.0[121.0,134.0]	128.0[121.0,134.0]	129.0[121.0,134.3]
Trimester 2 Median [Q1, Q3]	116.5[111.0,122.5]	118.0[112.0,124.5]	118.0[112.0,124.0]	119.0[112.6,126.0]
Trimester 3 Median [Q1, Q3]	119.0[112.0,125.5]	117.3[109.5,125.0]	117.0[110.0,125.0]	118.0[109.0,126.0]

*: 77 cases could not categorized into specific preterm subtype.

**Fig 2:**
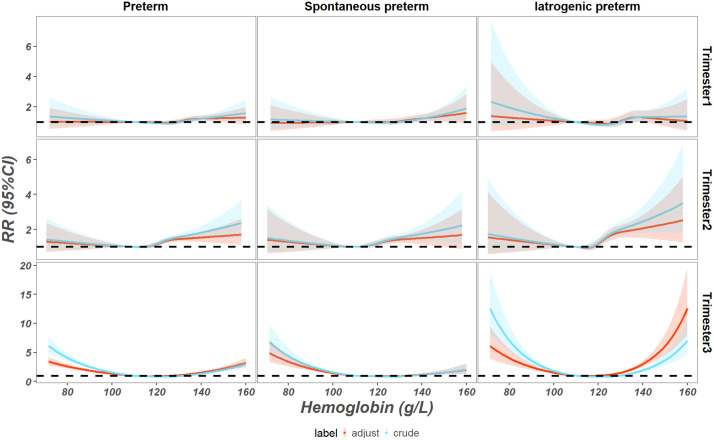
Association between overall hemoglobin concentration in pregnancy and preterm birth.

Regarding the association between Hb concentrations and preterm birth in the third trimester, a “L” shape of curve was observed in the association between spontaneous preterm and Hb concentration, and a “U” shape of curve was observed in the association between preterm, iatrogenic preterm and Hb concentration. In relation to an Hb cutoff value of 110 g/L, an Hb concentration >134 g/L in the first trimester or >119 g/L in the second trimester was associated with increased risk of preterm birth (risk ratio [RR] 1.16, 95% CI 1.00–1.34 and RR 1.12, 95% CI 1.00–1.26, respectively). In the third trimester, an Hb concentration <109 g/L or >130 g/L was associated with an increased risk of preterm birth (RR 1.00, 95% CI 1.00–1.01 and RR 1.08, 95% CI 1.00–1.17, respectively; **Fig 2**).

**Fig 2** also shows the association between Hb concentration levels and clinical subtypes of preterm birth. An Hb concentration level >138 g/L in the first trimester and >119 g/L in the second trimester was associated with an increased risk of spontaneous preterm birth (RR 1.21, 95% CI 1.00–1.47 and RR 1.16, 95% CI 1.00–1.35, respectively). In the third trimester, an Hb concentration level <109 g/L or >140 g/L was observed to be associated with an increased risk of spontaneous preterm birth (RR 1.00, 95% CI 1.00–1.01; and RR 1.18, 95% CI 1.00–1.39, respectively). An Hb concentration level >120 g/L (RR 1.23, 95% CI 1.01–1.49) in the second trimester was associated with an increased risk of iatrogenic preterm. Moreover, an Hb concentration level <109 g/L or >125 g/L in the third trimester was observed to be associated with an increased risk of iatrogenic preterm (RR 1.01, 95% CI 1.00–1.01 and RR 1.45, 95% CI 1.00–1.31, respectively). In the sensitivity analysis, the curves of association between Hb concentration levels and clinical subtypes of preterm birth were similar in the complete data and multiple imputation datasets (Supplementary Figure 1).

**Fig 3:**
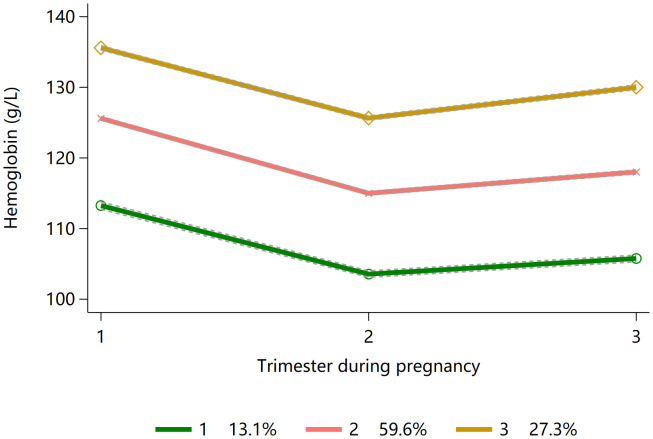
Trajectories of hemoglobin concentration from first to third trimester. Footnote: 1: Low, 2: Middle, 3: High.

We identified three groups of Hb concentration trajectories among 17,101 women in this study, as shown in **Fig 3**, including: (i) low (13.1%), (ii) middle (59.6%), and (iii) high (27.3%) concentration trajectory groups. These three trajectory groups showed a general parallel trend; the Hb concentration levels among all three groups showed a decreasing trend from the first to the second trimester and a slight increasing trend from the second to the third trimester. **Supplementary Table 2** in S1 File show the baseline characteristics and **Supplementary Table 6** in S1 File **showed** the sample size of the 17,101 women categorized according to gestational age and trajectory. The median values of Hb concentration (with the IQR) for those in consistently low, middle, and high Hb concentration groups were 113.0 g/L (108.0 g/L–118.0 g/L), 126.0 g/L (121.0 g/L–130.0 g/L), and 136.0 g/L (132.0 g/L–140.7 g/L), respectively, in the first trimester; 103.0 g/L (99.0 g/L–106.5 g/L), 115.0 g/L (111.0 g/L–119.0 g/L), and 126.0 g/L (122.0 g/L–130.0 g/L), respectively, in the second trimester; and 105.0 g/L (100.0 g/L–109.0 g/L), 118.0 g/L (113.5 g/L–122.0 g/L), and 130.0 g/L (126.0 g/L–135.0 g/L), respectively, in the third trimester. **Supplementary Table 3** shows the process of trajectory model selection.

We conducted an additional analysis to assess variations in preterm birth risk across distinct Hb trajectory groups (**Table 2**). Compared with the middle Hb concentration group, a constant high Hb concentration trajectory was associated with increased risk of preterm birth and iatrogenic preterm birth (odds ratio with its 95% Confidence Interval, OR[95%CI] were 1.22[1.05,1.42] and 1.35[1.03,1.78] respectively), and a constant low Hb concentration trajectory was associated with an increased risk of spontaneous preterm (OR[95%CI], 1.30[1.03,1.64]). In the sensitivity analysis, the results of association between Hb concentration trajectory group and clinical subtypes of preterm birth were similar in the complete data and multiple imputation datasets (Supplementary Table 4).

**Table 2:** Association between hemoglobin concentration trajectory group and preterm birth*.

Character	Hemoglobin Trajectories	Sample size	Preterm†	Spontaneous preterm‡	Iatrogenic preterm‡
**All**	Low	1958(11.45)	1.21[0.98,1.48]	1.30[1.03,1.64]	1.04[0.69,1.58]
	Middle	10677(62.43)	ref	ref	ref
	High	4466(26.12)	1.22[1.05,1.42]	1.20[1.00,1.43]	1.35[1.03,1.78]
28-31 weeks	Low	12(17.65)	1.59[0.79,2.98]	2.13[1.01,4.49]	0.90[0.20,4.15]
	Middle	40(58.82)	ref	ref	ref
	High	16(23.53)	0.81[0.43,1.44]	0.63[0.27,1.47]	1.21[0.48,3.04]
32-36 weeks	Low	107(12.60)	1.18[0.95,1.46]	1.24[0.97,1.59]	1.06[0.69,1.62]
	Middle	486(57.24)	ref	ref	ref
	High	256(30.15)	1.26[1.07,1.47]	1.24[1.03,1.49]	1.36[1.01,1.81]
**Maternal age**					
<35 yrs	Low	1622(11.41)	1.18[0.93,1.49]	1.22[0.93,1.60]	1.15[0.71,1.88]
	Middle	8878(62.48)	ref	ref	ref
	High	3710(26.11)	1.20[1.00,1.43]	1.15[0.94,1.42]	1.43[1.02,1.99]
≥35 yrs	Low	336(11.63)	1.32[0.86,1.96]	1.62[1.00,2.60]	0.88[0.41,1.89]
	Middle	1798(62.24)	ref	ref	ref
	High	755(26.13)	1.29[0.95,1.75]	1.34[0.92,1.94]	1.22[0.74,2.03]
**Maternal education**					
Below college	Low	317(12.84)	1.19[0.73,1.88]	1.17[0.64,2.13]	1.39[0.67,2.89]
	Middle	1570(63.61)	ref	ref	ref
	High	581(23.54)	1.34[0.93,1.90]	1.38[0.88,2.16]	1.28[0.71,2.30]
Above college	Low	1285(12.49)	1.31[1.01,1.67]	1.40[1.06,1.86]	1.09[0.63,1.89]
	Middle	6507(63.25)	ref	ref	ref
	High	2496(24.26)	1.14[0.93,1.40]	1.21[0.95,1.52]	1.08[0.71,1.63]
**Parity**					
Primipara	Low	1112(9.78)	1.13[0.84,1.50]	1.07[0.76,1.50]	1.23[0.69,2.20]
	Middle	7037(61.91)	ref	ref	ref
	High	3218(28.31)	1.31[1.08,1.58]	1.26[1.01,1.56]	1.48[1.02,2.14]
Multipara	Low	838(14.74)	1.33[0.99,1.77]	1.64[1.18,2.29]	0.88[0.48,1.60]
	Middle	3614(63.56)	ref	ref	ref
	High	1234(21.70)	1.12[0.86,1.44]	1.09[0.79,1.51]	1.26[0.82,1.93]
**Previous complication**					
	Low	369(11.92)	1.49[0.95,2.26]	1.53[0.92,2.54]	1.55[0.72,3.34]
	Middle	1855(59.92)	ref	ref	ref
	High	872(28.17)	1.14[0.81,1.59]	1.10[0.74,1.65]	1.30[0.72,2.35]
**Previous preterm**					
	Low	27(14.06)	3.74[1.16,11.87]	8.70[2.08,36.38]	0.88[0.07,10.51]
	Middle	105(54.69)	ref	ref	ref
	High	60(31.25)	1.43[0.56,3.59]	1.84[0.58,5.81]	1.57[0.31,7.90]
**Scarred uterus**					
	Low	365(14.01)	1.35[0.87,2.04]	1.69[1.01,2.84]	0.97[0.47,2.02]
	Middle	1650(63.32)	ref	ref	ref
	High	591(22.68)	1.11[0.77,1.59]	1.09[0.67,1.77]	1.22[0.72,2.07]
**Pre-eclampsia**					
	Low	21(4.41)	1.24[0.35,3.77]	0.53[0.04,6.44]	1.51[0.41,5.64]
	Middle	238(50.00)	ref	ref	ref
	High	217(45.59)	1.34[0.82,2.21]	1.25[0.54,2.91]	1.47[0.83,2.60]
**Gestational hypertension**					
	Low	31(5.55)	3.16[0.71,11.3]	2.27[0.3,17.12]	3.38[0.49,23.30]
	Middle	273(48.84)	ref	ref	ref
	High	255(45.62)	1.28[0.56,2.99]	2.00[0.69,5.83]	0.37[0.07,1.87]
**Gestational diabetes**					
	Low	340(8.75)	1.03[0.62,1.63]	1.15[0.68,1.93]	0.64[0.19,2.10]
	Middle	2278(58.61)	ref	ref	ref
	High	1269(32.65)	1.30[0.99,1.71]	1.08[0.77,1.50]	2.04[1.25,3.34]

*: ORs presented in Table 2 were adjusted for factors as followed: Medical history; Maternal nationality; Maternal work; Maternal education; Maternal age; Parity; Fetal gender; Preterm history; Scarred uterus; Assisted reproduction; Cervical incompetence;

†: adjusted Odds Ratio, showed as aOR[95%CI];

‡: adjusted Odds Ratio with Bonferroni adjustment, showed as aOR[95%CI];

In subgroup analysis, a constant high Hb concentration trajectory was associated with increased risk of preterm birth, iatrogenic preterm birth and spontaneous preterm among primipara women (OR[95%CI] were 1.31[1.08,1.58], 1.26[1.01,1.56] and 1.48[1.02,2.14] respectively). A constant low Hb concentration trajectory was associated with increased risk of preterm birth and spontaneous preterm among women with a previous preterm birth (OR[95%CI] were 3.74[1.16,11.87] and 8.70[2.08,36.38]). Moreover, a constant high Hb concentration level was associated with an increased risk of iatrogenic preterm birth among women diagnosed with gestational diabetes (OR[95%CI], 2.04[1.25,3.34]; **Table 2**).

## Discussion

Hb concentration tests are commonly performed in antenatal care. Our findings indicated that both high and low Hb concentration levels were associated with an increased risk of preterm birth. A low Hb concentration level was found to be associated with an increased risk of spontaneous and iatrogenic preterm births in late pregnancy, and a high Hb concentration level was associated with an increased risk of iatrogenic preterm births in middle and late pregnancy. Longitudinal Hb concentration levels showed three parallel flat “U” trajectories in pregnancy. A consistently high Hb concentration level was associated with an increased risk of preterm birth and iatrogenic preterm births, and a consistently low Hb concentration level was associated with an increased risk of spontaneous preterm birth. This is the first multicenter observational study to simultaneously investigate the association between preterm birth with its clinical subtypes and Hb concentration measured at a point in time and its longitudinal trajectory.

We observed an association between a low Hb concentration level and an increased risk of preterm birth with its clinical subtypes in late pregnancy, which is consistent with findings reported in previous studies. However, unlike in previous studies, we did not find this association prior to the third trimester [6,17,18]. A low Hb concentration level is considered to be closely associated with iron deficiency, which is the main cause of anemia worldwide. The association between iron deficiency anemia and preterm birth can be explained through the potential mechanisms of hypoxia and infection [19,20]. Chronic hypoxia from anemia could initiate a stress response, stimulate placental corticotrophin-releasing hormone and increased cortisol production by the fetus, and result in early delivery. Moreover, iron deficiency may increase the risk of preterm birth due to maternal infections through altering T and B cell proliferation, reducing the killing activity of phagocytes and neutrophils, and decreasing bactericidal and natural killer cell activity [21–23]. Pregnant women are particularly vulnerable to iron deficiency anemia owing to their high iron demand during pregnancy [24,25]. In early pregnancy, with a net saving of Hb (due to the cessation of menstruation) and a low iron requirement, maternal Hb concentrations are likely to sustain the development of fetal and placenta growth. However, as pregnancy progresses, iron requirements markedly increase to a maximum of 3–8 mg iron per day during the third trimester [26,27]. Excess iron requirements and resulting iron deficiency may stimulate fetal hypoxia or maternal infection, and are likely to result in an increased risk of preterm birth in late pregnancy. Moreover, the non-significant association between Hb concentration and preterm birth in our study may be related to more medically related confounders adjusted in the analysis. Previous studies have shown that preterm birth may be due to several potential mechanisms simultaneously, and that some other potential risk factors, such as utero-placental ischemia or uterine over-distension, may be associated with an increased risk of preterm birth [9,28]. Previous studies have found that women with a history of caesarean section had a lower uterine artery volume blood flow and a lower maternal cardiac output fraction distributed to the uteroplacental circulation [29]. Thus, it is possible to infer that a scarred uterus increases the risk of uteroplacental ischemia. Additionally, a scarred uterus is associated with an increased risk of uterine rupture, which may indicate restricted uterine dilation [30]. In contrast with previous studies, we also adjusted for a scarred uterus as a confounder in the analysis, which may have distinguished the effect of uteroplacental ischemia and uterine over-distension from the overall risk of preterm birth and influenced the non-significant association found between Hb concentration and preterm birth to some extent [31–35].

We found that an increased risk of preterm birth was associated with an increase in Hb concentration levels in the second and third trimesters, which is consistent with findings of a previous study [6]. We additionally found a steeper increase in iatrogenic preterm births than in spontaneous preterm births, with Hb levels increasing in the second and third trimesters. This phenomenon may partly be explained by the association between higher Hb levels and an increased risk of maternal complications, such as pre-eclampsia, gestational hypertension, and gestational diabetes, which are also risk factors for iatrogenic preterm birth [36]. We confirmed our hypothesis by curves of association between Hb concentration levels and preterm in the subgroup of women diagnosed with pre-eclampsia, gestational hypertension and gestational diabetes separately. We found that among women diagnosed with pre-eclampsia and gestational diabetes were on an increasing risk of iatrogenic preterm birth when Hb concentration levels increasing in the third trimester (Supplementary figure 2). Thus, the increased risk of preterm birth in pregnant women with high Hb concentration levels may be enhanced in cases of iatrogenic preterm birth; therefore, pregnant women with high Hb concentrations during middle or late pregnancy should be informed in relation to potential maternal complications and an increased risk of iatrogenic preterm birth.

In this study, we found that the association between Hb concentration levels and preterm birth differed in separate trimesters. However, these associations were cross-sectional conclusions based on point-of-time measurements, and the association between Hb concentration levels in different trimesters and preterm birth remains unknown. It has previously been established that maternal Hb concentration levels change during pregnancy, thus, it is crucial to investigate this association between preterm birth and changes in maternal Hb concentration longitudinally. Some researchers have investigated this association through setting certain cutoff values to categorize the continuous Hb concentration levels into separate sub-categories, such as anemia, and then evaluating the Hb change in different groups [5,18,37]. However, the upper limit value for adverse effects used in those studies is not internationally recognized, as is the case with anemia, but omitting the upper limit for Hb concentration may result in a biased conclusion. We adopted a longitudinal data-driven approach rather than a simple cutoff value to evaluate increase or decrease of Hb during pregnancy. We identified three parallel “U” shaped Hb trajectories in pregnancy, and found that the flat “U” shape was consistent with the physiological changes associated with pregnancy [12,13,37]. We also found that the Hb concentration levels in each group stayed relatively stable. While Hb concentration levels may change during pregnancy, women with high Hb concentrations retain a relatively high level until late pregnancy, and women with lower Hb concentration levels maintain a relatively constant low level.

An association was observed in terms of heterogeneity between clinical preterm subtypes and the Hb concentration trajectory. Pregnant women with a constant high Hb concentration level should be treated differently due to their different risks of preterm birth or its clinical subtypes. We found that a consistently high Hb level was associated with an increased risk of iatrogenic preterm birth, and a consistently low Hb level was associated with an increased risk of spontaneous preterm birth. While previous research has attributed the increased risk of preterm birth at high Hb concentration levels to increased blood viscosity which may related to adverse placental conditions, resulting in a lack of hemodilution and reduced placental perfusion [5,38–40], and attributed the increased risk of preterm birth at low Hb concentration levels to iron deficiency, maternal infection, hypoxia and oxidative stress [7], rare study confirmed the association between constant high or low Hb concentration levels and clinical preterm subtypes. Given the difference in the association between Hb concentration levels and the clinical subtypes of preterm birth, we consider that heterogeneity may exist in the mechanism involved in spontaneous and iatrogenic preterm births related to Hb concentration. Future research needs to focus on the mechanisms involved in high Hb levels and preterm birth.

In subgroup analysis, we found a constant low Hb concentration level among women with a scarred uterus were in association with increased risk of spontaneous preterm. A history of previous caesarean section was found to be associated with lower uterine artery volume blood flow (356.26 ± 213.72 ml/minute vs. 456.41 ± 209.70 ml/minute, *P* = 0.038) and higher uterine vascular resistance (0.32 ± 0.20 vs. 0.22 ± 0.14, *P* = 0.026) [29]. With the superimposed effect of low Hb concentration level, aggravated hypoxia and oxidative stress may increase the risk of spontaneous preterm birth. In addition, we also found constant high Hb concentration level was associated with preterm birth within its clinical subtypes among primiparas. Considering lower maternal cardiac adaption of plasma volume expansion among primiparas [41,42], higher Hb concentration level may increase blood viscosity and finally increase risk of preterm birth.

This study had several limitations. Firstly, the original study was not designed to investigate preterm birth, and that it does not include births before 28 weeks. Besides, hematocrit values were not measured, so the potential mechanism between Hb concentration and preterm birth was based on previous research. The mechanism of iatrogenic preterm birth owing to blood viscosity should be explored further with consideration of placental blood volume. Iron supplementation was not adjusted for as a confounder in the model analysis. While information on folic acid supplementation was collected, we did not adjust for its effect in the analysis because of the large proportion of missing values. And variables of maternal education and maternal nationality had significant proportions of missing data which may also had potential effect on the analysis results. Furthermore, due to sample size limitations some real differences may not have reached statistical significance (especially some subgroup analysis results). Further studies can explore the results with marginal correlation by expanding sample size and statistical power.

## Conclusion

This is the first observational multicenter study to simultaneously assess the association between clinical subtypes of preterm birth and Hb concentration levels at separate time points and longitudinal trajectories. Both low and high Hb concentration levels were associated with an increased risk of late preterm birth, and women with high Hb concentration levels need to be informed of the increased risk of iatrogenic preterm births in middle and late pregnancy. The parallel “U” shape found in Hb trajectories indicated that women with high Hb concentration levels maintained a relatively high level until late pregnancy. A consistently high Hb concentration level was associated with an increased risk of iatrogenic preterm, and a consistently low Hb concentration level was associated with an increased risk of spontaneous preterm. Thus, pregnant women with either low or high Hb concentration levels in early pregnancy should be made aware of the potential risk of preterm birth.

## Supporting information

S1 File Supplementary file. Contains supplementary results of this study.
(PDF)

S1 Code Contains the code for regular expression in this study. (PDF)
