# Supplementary material for: Association between maternal hemoglobin concentration levels and preterm birth clinical subtypes: A retrospective, observational, multicenter study
Source: PLoS One. 2026 May 13;21(5):e0348071. doi: 10.1371/journal.pone.0348071 (PMC13170862; doi:10.1371/journal.pone.0348071)
Supplement: S1 File — Contains supplementary results of this study. (PDF) [file pone.0348071.s001.pdf]

Supplementary file:

Supplementary Table 1. Demographic character distribution of complete datasets and incomplete datasets

**Character**	**RCS data**		**GBTM data**	
	complete data	incomplete data	complete data	incomplete data
	N=32319	N=39820	N=6326	N=10775
**Preterm**				
no	29388(91.93)	37898(94.34)	5877(93.88)	10282(94.84)
yes	2581(8.07)	2272(5.66)	383(6.12)	559(5.16)
**Preterm subtype**				
no	29388(91.93)	37898(94.34)	5877(93.88)	10282(94.84)
Iatrogenic preterm	1084(3.39)	723(1.80)	138(2.20)	119(1.10)
Spontaneous preterm	1456(4.55)	1513(3.77)	238(3.80)	427(3.94)
Unknown	41(0.13)	36(0.09)	7(0.11)	13(0.12)
**Maternal age**				
<35 yrs	25953(81.18)	33906(84.45)	5006(79.97)	9204(84.92)
≥35 yrs	6016(18.82)	6241(15.55)	1254(20.03)	1635(15.08)
Unknown		23		2
**Parity**				
Primipara	19866(62.14)	24316(60.96)	4190(66.93)	7177(66.50)
Multipara	12103(37.86)	15571(39.04)	2070(33.07)	3616(33.50)
Unknown		283		48
**Maternal nationality**				
Han	31534(98.64)	22573(99.30)	6153(98.29)	4837(99.36)
Other	435(1.36)	160(0.70)	107(1.71)	31(0.64)
Unknown		17437		5973
**Maternal work**				
Manual work	4477(14.00)	2304(8.90)	834(13.32)	562(8.86)
Non-manual work	21337(66.74)	13079(50.51)	4435(70.85)	3051(48.12)
other	6155(19.25)	10509(40.59)	991(15.83)	2728(43.02)
Unknown		14278		4500
**Maternal education**				
below college	8363(26.16)	3567(17.36)	1538(24.57)	930(14.32)
College and above	23606(73.84)	16985(82.64)	4722(75.43)	5566(85.68)
Unknown		19618		4345
**Medical history**				
None	26754(83.69)	34228(85.22)	5041(80.53)	8963(82.68)
At least one	5215(16.31)	5938(14.78)	1219(19.47)	1877(17.32)
Unknown		4		1
**Preterm history**				
None	31521(98.60)	38859(98.89)	6174(98.63)	10586(99.01)
Yes	448(1.40)	437(1.11)	86(1.37)	106(0.99)
Unknown		874		149
**Scarred uterus**				
No	25910(81.05)	33113(82.48)	5208(83.19)	9283(85.66)
Yes	6059(18.95)	7035(17.52)	1052(16.81)	1554(14.34)

Unknown		22		4
**Fetal gender**				
Boy	16675(52.16)	20653(52.05)	3273(52.28)	5565(51.79)
Girl	15294(47.84)	19024(47.95)	2987(47.72)	5181(48.21)
Unknown		493		95
**Assisted reproduction**				
No	30388(95.05)	38837(96.73)	5618(89.74)	10437(96.31)
Yes	1581(4.95)	1311(3.27)	642(10.26)	400(3.69)
Unknown		22		4
**Cervical incompetence**				
No	31759(99.34)	40013(99.66)	6211(99.22)	10810(99.75)
Yes	210(0.66)	135(0.34)	49(0.78)	27(0.25)
Unknown		22		4

Analysis based on complete data

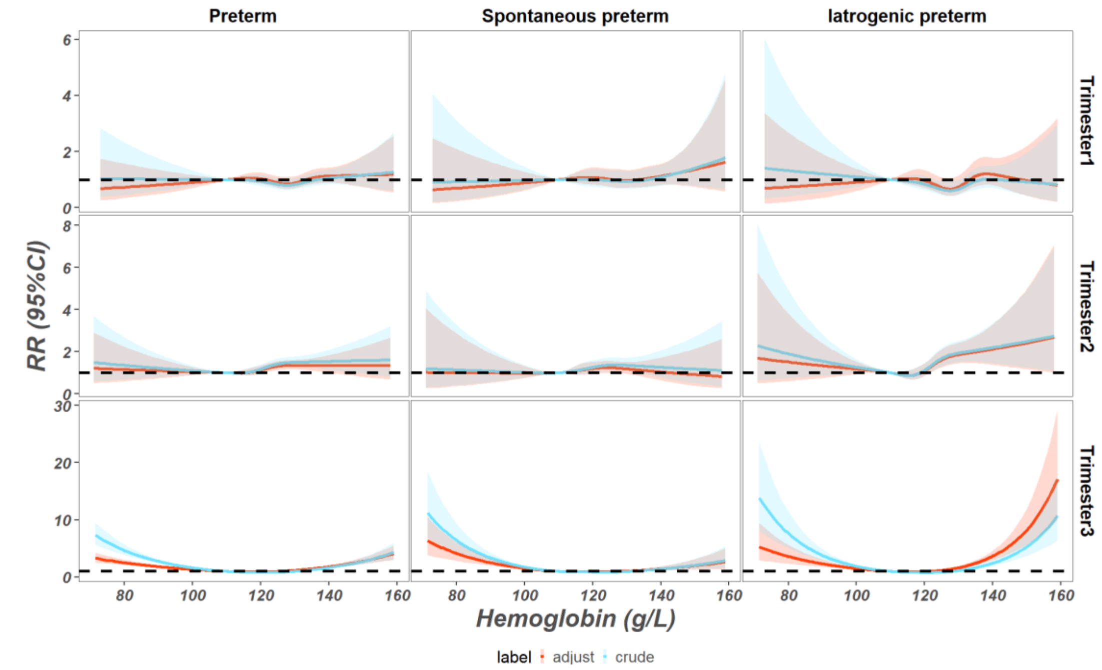

Multiple imputation (polyregression)

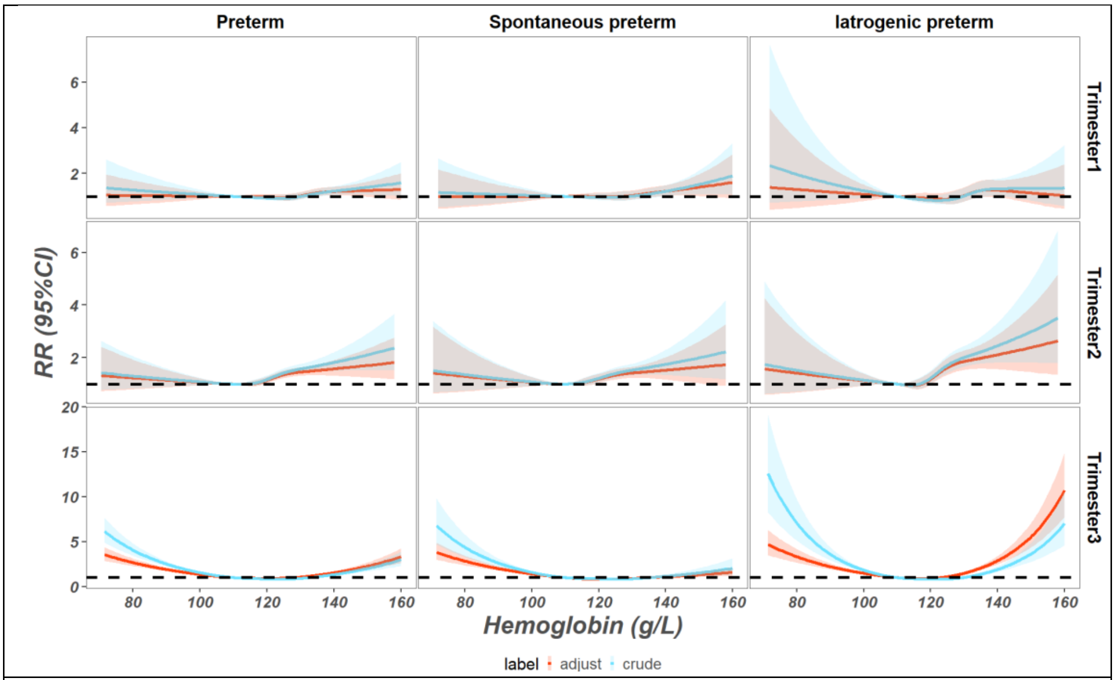

Multiple imputation (random forest)

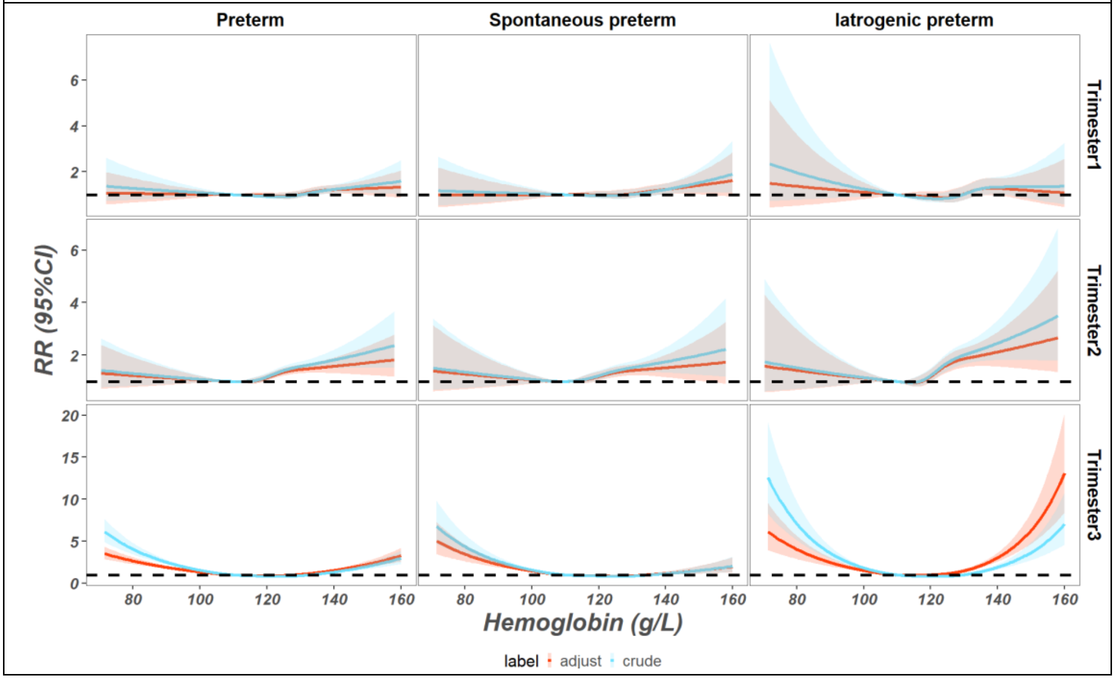

Supplementary Figure 1. Sensitive analysis of association between overall hemoglobin concentration in pregnancy and preterm birth

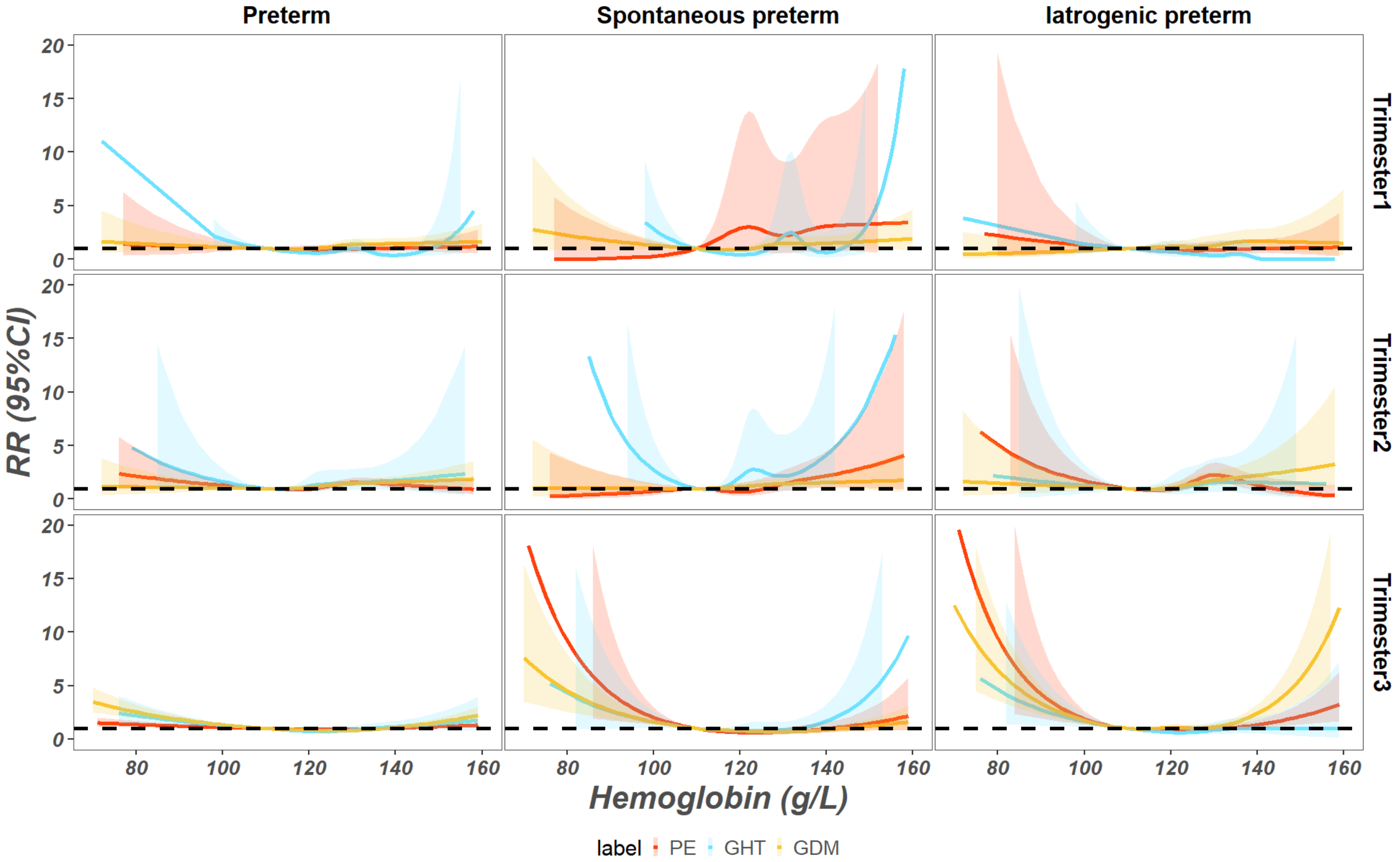

Supplementary Figure 2. Association between overall hemoglobin concentration in pregnancy and preterm birth in different subgroups

PE: pre-eclampsia; GHT: gestational hypertension; GDM: gestational diabetes;

Supplementary Table2. Baseline character for trajectory analysis pregnant women (N=17101)

Character	Term birth	Preterm birth	Subtype of preterm birth*	
			Spontaneous Preterm	Iatrogenic preterm
	N=16159	N=942	N=665	N=257
**Maternal age**				
<35 yrs	13509(83.60%)	701(74.42%)	508(76.39%)	176(68.48%)
above 35 yrs	2648(16.39%)	241(25.58%)	157(23.61%)	81(31.52%)
Unknown	2(0.01%)			
**Parity**				
Primipara	10809(66.89%)	558(59.24%)	413(62.11%)	137(53.31%)
Multipara	5311(32.87%)	375(39.81%)	249(37.44%)	116(45.14%)
Unknown	39(0.24%)	9(0.96%)	3(0.45%)	4(1.56%)
**Medical history**				
None	13263(82.08%)	741(78.66%)	528(79.40%)	197(76.65%)
At least one	2895(17.92%)	201(21.34%)	137(20.60%)	60(23.35%)
Unknown	1(0.01%)			
**Preterm history**				
None	15866(98.19%)	894(94.90%)	632(95.04%)	244(94.94%)
Yes	157(0.97%)	35(3.72%)	24(3.61%)	10(3.89%)
Unknown	136(0.84%)	13(1.38%)	9(1.35%)	3(1.17%)
**Scarred uterus**				
No	13736(85.01%)	755(80.15%)	559(84.06%)	180(70.04%)
Yes	2420(14.98%)	186(19.75%)	106(15.94%)	77(29.96%)
Unknown	3(0.02%)	1(0.11%)		
**Fetal gender**				
Boy	8294(51.33%)	544(57.75%)	376(56.54%)	154(59.92%)
Girl	7791(48.21%)	377(40.02%)	275(41.35%)	96(37.35%)
Unknown	74(0.46%)	21(2.23%)	14(2.11%)	7(2.72%)
**Assisted reproduction**				
No	15201(94.07%)	854(90.66%)	607(91.28%)	228(88.72%)
Yes	955(5.91%)	87(9.24%)	58(8.72%)	29(11.28%)
Unknown	3(0.02%)	1(0.11%)		
**Cervical incompetence**				
No	16095(99.60%)	926(98.30%)	658(98.95%)	249(96.89%)
Yes	61(0.38%)	15(1.59%)	7(1.05%)	8(3.11%)
Unknown	3(0.02%)	1(0.11%)		
**Pre-eclampsia**				
No	15467(97.11%)	1000(79.37%)	732(95.56%)	244(54.22%)
Yes	355(2.23%)	229(18.17%)	30(3.92%)	199(44.22%)
Unknown	105(0.66%)	31(2.46%)	4(0.52%)	7(1.56%)
**Gestational hypertension**				
No	15468(97.12%)	1203(95.48%)	751(98.04%)	428(95.11%)
Yes	354(2.22%)	26(2.06%)	11(1.44%)	15(3.33%)
Unknown	105(0.66%)	31(2.46%)	4(0.52%)	7(1.56%)
**Gestational diabetes**				
No	12377(77.71%)	943(74.84%)	577(75.33%)	342(76.00%)

Yes	3445(21.63%)	286(22.70%)	185(24.15%)	101(22.44%)
Unknown	105(0.66%)	31(2.46%)	4(0.52%)	7(1.56%)
**Hemoglobin concentration (g/L)**				
Trimester 1 Median [Q1, Q3]	127.0[121.0,133.0]	128.0[121.0,134.0]	127.0[121.0,134.0]	129.0[121.5,134.0]
Trimester 2 Median [Q1, Q3]	116.0[110.5,122.0]	118.0[111.0,124.0]	118.0[111.0,124.0]	119.5[112.7,125.0]
Trimester 3 Median [Q1, Q3]	120.0[113.0,126.5]	119.0[111.7,126.0]	118.8[112.0,126.0]	120.0[111.5,126.5]

*: 20 cases could not be categorized in to specific preterm subtype

Supplementary Table 3. Average posterior probability of group assignment and Bayesian information criterion (BIC) statistics of group-based trajectory models (GBTM) for hemoglobin concentration

Group	Model	AvepP%	Proportion per class%	BIC	d-BIC	Ej
2	Linear	86.37-88.42	43.86-56.14	-202779	3.29	0.593
	Quadratic	88.81-90.20	45.07-54.93	-198592	4.40	0.655
3	Linear	83.84-86.85-83.58	10.00-62.29-27.72	-201297	4.94	0.696
	Quadratic	**86.46-88.38-86.48**	**12.79-60.05-27.16**	**-196290**	**6.59**	**0.736**
4	Linear	89.78-82.03-83.16-81.77	1.64-28.71-56.55-13.11	-200698	6.59	0.710
	Quadratic	86.92-84.58-84.94-85.00	3.19-32.96-51.18-12.68	-195275	8.79	0.745
5	Linear	89.96-78.81-77.95-77.90-79.70	1.00-13.70-48.69-32.84-3.76	-200495	8.24	0.686
	Quadratic	82.67-92.25-81.07-81.56-83.11	3.41-0.59-32.98-50.20-12.81	-194725	10.99	0.786

Supplementary Table 4. Sensitive analysis of association between hemoglobin concentration trajectory group and preterm birth

	Hemoglobin Trajectories	Preterm†	Spontaneous preterm‡ (2)	Iatrogenic preterm‡ (1)
**Analysis based on complete data**	Low	1.39 [1.01,1.88]	1.40[0.95,2.06]	1.47[0.90,2.41]
	Middle	ref	ref	ref
	High	1.33[1.04,1.69]	1.37[1.02,1.85]	1.28[0.86,1.90]
**Multiple imputation (polyregression)**	Low	1.24[1.00,1.51]	1.33[1.05,1.68]	1.05[0.69,1.58]
	Middle	ref	ref	ref
	High	1.20[1.03,1.40]	1.18[0.99,1.42]	1.33[1.01,1.74]
**Multiple imputation (random forest)**	Low	1.23[1.00,1.51]	1.33[1.06,1.68]	1.03[0.69,1.56]
	Middle	ref	ref	ref
	High	1.20[1.03,1.40]	1.18[0.99,1.41]	1.33[1.01,1.75]

Supplementary Table 5. Association between change of Hb concentration and preterm birth*

Trimester #	Hemoglobin Trajectories Group	Preterm†	Spontaneous preterm‡	Iatrogenic preterm‡
**T1-T2**	All	1.02[1.01,1.03]	1.01[1.00,1.03]	1.03[1.01,1.04]
	Low	1.01[0.99,1.03]	1.01[0.98,1.03]	1.03[1.00,1.07]
	Middle	1.02[1.00,1.03]	1.02[1.00,1.03]	1.02[1.00,1.05]
	High	1.01[0.99,1.03]	1.01[0.98,1.03]	1.03[0.99,1.06]
**T2-T3**	All	0.96[0.95,0.97]	0.96[0.95,0.97]	0.96[0.94,0.98]
	Low	0.94[0.92,0.97]	0.96[0.93,0.98]	0.91[0.86,0.95]
	Middle	0.96[0.94,0.97]	0.95[0.94,0.97]	0.95[0.93,0.98]
	High	0.98[0.96,1.00]	0.98[0.95,1.00]	0.99[0.96,1.03]

#: T1, the first trimester; T2, the second trimester; T3, the third trimester;

*: Hb change rate (T1–T2) = (Hb in T2 − Hb in T1) / Hb in T1 × 100%

Hb change rate (T2–T3) = (Hb in T3 − Hb in T2) / Hb in T2 × 100%

Supplementary Table 6. Sample size of subgroups in each Hb trajectory group

	Hb Trajectories	Full term	Preterm†	Spontaneous preterm‡	Iatrogenic preterm‡
**All**	Low	1835(11.36%)	123(13.06%)	93(13.98%)	28(10.89%)
	Middle	10136(62.73%)	541(57.43%)	381(57.29%)	145(56.42%)
	High	4188(25.92%)	278(29.51%)	191(28.72%)	84(32.68%)
28-31 weeks	Low	1839(11.36%)	12(17.65%)	10(23.81%)	2(8.70%)
	Middle	10151(62.72%)	40(58.82%)	25(59.52%)	12(52.17%)
	High	4194(25.91%)	16(23.53%)	7(16.67%)	9(39.13%)
32-36 weeks	Low	1839(11.36%)	107(12.60%)	80(13.27%)	25(10.92%)
	Middle	10151(62.72%)	486(57.24%)	344(57.05%)	130(56.77%)
	High	4194(25.91%)	256(30.15%)	179(29.68%)	74(32.31%)
**Maternal age**					
<35 yrs	Low	1531(11.33%)	91(12.98%)	69(13.58%)	20(11.36%)

	Middle	8472(62.71%)	406(57.92%)	297(58.46%)	96(54.55%)
	High	3506(25.95%)	204(29.10%)	142(27.95%)	60(34.09%)
≥35 yrs	Low	304(11.48%)	32(13.28%)	24(15.29%)	8(9.88%)
	Middle	1663(62.80%)	135(56.02%)	84(53.50%)	49(60.49%)
	High	681(25.72%)	74(30.71%)	49(31.21%)	24(29.63%)
**Maternal education**					
Below college	Low	293(12.78%)	24(13.64%)	14(13.33%)	10(15.38%)
	Middle	1469(64.09%)	101(57.39%)	60(57.14%)	37(56.92%)
	High	530(23.12%)	51(28.98%)	31(29.52%)	18(27.69%)
Above college	Low	1201(12.34%)	84(15.16%)	66(15.98%)	16(12.60%)
	Middle	6182(63.51%)	325(58.66%)	237(57.38%)	77(60.63%)
	High	2351(24.15%)	145(26.17%)	110(26.63%)	34(26.77%)
**Parity**					
Primipara	Low	1055(9.76%)	57(10.22%)	41(9.93%)	14(10.22%)
	Middle	6723(62.20%)	314(56.27%)	237(57.38%)	73(53.28%)
	High	3031(28.04%)	187(33.51%)	135(32.69%)	50(36.50%)
Multipara	Low	773(14.55%)	65(17.33%)	52(20.88%)	13(11.21%)
	Middle	3393(63.89%)	221(58.93%)	142(57.03%)	70(60.34%)
	High	1145(21.56%)	89(23.73%)	55(22.09%)	33(28.45%)
**Previous complication**					
	Low	339(11.71%)	30(14.93%)	21(15.33%)	9(15.00%)
	Middle	1744(60.24%)	111(55.22%)	76(55.47%)	32(53.33%)
	High	812(28.05%)	60(29.85%)	40(29.20%)	19(31.67%)
**Previous preterm**					
	Low	20(12.74%)	7(20.00%)	6(25.00%)	1(10.00%)
	Middle	89(56.69%)	16(45.71%)	10(41.67%)	5(50.00%)
	High	48(30.57%)	12(34.29%)	8(33.33%)	4(40.00%)
**Scarred uterus**					
	Low	335(13.84%)	30(16.13%)	21(19.81%)	9(11.69%)
	Middle	1541(63.68%)	109(58.60%)	60(56.60%)	46(59.74%)
	High	544(22.48%)	47(25.27%)	25(23.58%)	22(28.57%)
**Pre-eclampsia**					
	Low	16(4.26%)	5(5.00%)	1(3.57%)	4(5.56%)
	Middle	192(51.06%)	46(46.00%)	14(50.00%)	32(44.44%)
	High	168(44.68%)	49(49.00%)	13(46.43%)	36(50.00%)
**Gestational hypertension**					
	Low	27(5.10%)	4(13.33%)	2(10.53%)	2(18.18%)
	Middle	261(49.34%)	12(40.00%)	6(31.58%)	6(54.55%)
	High	241(45.56%)	14(46.67%)	11(57.89%)	3(27.27%)
**Gestational diabetes**					
	Low	319(8.78%)	21(8.24%)	18(9.89%)	3(4.11%)
	Middle	2144(59.03%)	134(52.55%)	101(55.49%)	33(45.21%)
	High	1169(32.19%)	100(39.22%)	63(34.62%)	37(50.68%)
