# Supplementary material for: Association between maternal hemoglobin concentration levels and preterm birth clinical subtypes: A retrospective, observational, multicenter study
Source: PLoS One. 2026 May 13;21(5):e0348071. doi: 10.1371/journal.pone.0348071 (PMC13170862; doi:10.1371/journal.pone.0348071)
Supplement: S1 Code — (PDF) [file pone.0348071.s002.pdf]

```
data dta_cov;
    set dta11;
    length comb all $500.;
    comb=compress(var403||var404||var405||var406||var407||var408||var409||var410
||var411||var412||var413||var414||
    var415||var416||var417||var418||var419||var420||var421||var422||var423||var4
24);
    all=compress(var393||var394||var395||var396||var397);;

    if find(upcase(compress(all)),"G0")>0 or find(upcase(compress(all)),"孕0")>0
then parity=0;
    else if find(upcase(compress(all)),"G0")=0 and find(upcase(all),"孕0")=0 then
do;
    text1=prxchange("s/(.+)?(G\d+P\d+)(.+)?/\2/",-1,strip(compress(all)));
    text2=prxchange("s/^(G\d+P)(\d+)/\2/",-1,strip(text1));
    text3=prxchange("s/(.+)?(孕\d+产\d+)(.+)?/\2/",-1,strip(compress(all)));
    text4=prxchange("s/^(孕\d+产)(\d+)/\2/",-1,strip(text3));
    if  .  lt  input(text2,best.)  le  10  then  parity1=input(text2,best.);else
parity1=input(text4,best.);
    if parity1=. then do;
    text_1=prxchange("s/(.+)?(\d+-\d+-\d+-\d+)(.+)?/\2/",-1,strip(compress(all))
);
    text_2_temp = prxchange("s/^(\d+)-(\d+)-\d+-\d+/\1 \2/", -1, strip(text_1));
    term_preterm_sum     =     sum(input(scan(text_2_temp,     1,     "     "),
best.),input(scan(text_2_temp, 2, " "), best.));
    parity1 = term_preterm_sum;

    end;

    text5=prxchange("s/(.+)?(G\d+P\d+)(.+)?/\2/",-1,strip(compress(comb)));
    text6=prxchange("s/^(G\d+P)(\d+)/\2/",-1,strip(text5));
    text7=prxchange("s/(.+)?(孕\d+产\d+)(.+)?/\2/",-1,strip(compress(comb)));
    text8=prxchange("s/^(孕\d+产)(\d+)/\2/",-1,strip(text7));
    if  .  lt  input(text6,best.)  le  10  then  parity2=input(text6,best.);else
parity2=input(text8,best.);
    if parity2=. then do;
    text_1=prxchange("s/(.+)?(产\d+次)(.+)?/\2/",-1,strip(compress(comb)));
    text_2=substr(text_1,3,1);
    parity2=input(text_2,best.);
    end;

    if parity=. and parity1 ne . then parity=parity1;
    else if parity=. and parity1=. and parity2 ne 0 then parity=parity2-1;
    else if parity=. and parity1=. and parity2=0 then parity=0;
```

```
    end;

    keep idd_ord comb all parity ;
run;
```
